# Non-motor correlates of anxiety in *de novo* Parkinson’s disease: a cross-sectional study with exploratory subgroup analyses

**DOI:** 10.3389/fnins.2026.1955684

**Published:** 2026-09-25

**Authors:** Guixiang He, Gendi Wang, Haihua Sun, Shufang Wang, Xia Chen, Weiguo Liu, Pinglei Pan

**Affiliations:** 1 Department of Neurology, Affiliated Hospital 6 of Nantong University, Yancheng Third People’s Hospital, Yancheng, China; 2 Department of Neurology, Affiliated Nanjing Brain Hospital, Nanjing Medical University, Nanjing, China

**Keywords:** anxiety, clinical correlates, *de novo*, non-motor symptoms, Parkinson’s disease, subgroup analysis

## Abstract

**Objective:**

Anxiety is a prevalent early non-motor symptom of Parkinson’s disease (PD), but its clinical correlates remain poorly defined in *de novo*, drug-naïve patients. This retrospective cross-sectional study enrolled 284 medication-naïve *de novo* PD patients and 147 age- and sex-matched healthy controls to identify non-motor correlates of anxiety after adjustment for demographic and motor covariates, and explore potential subgroup heterogeneity.

**Methods:**

Anxiety was assessed with the Hamilton Anxiety Rating Scale; multivariate logistic regression and prespecified stratified analyses by age, sex, disease duration, Hoehn-Yahr stage and motor severity were performed.

**Results:**

Clinically significant anxiety was detected in 44.0% of PD patients versus 8.1% of controls (*p* < 0.001), with psychic anxiety as the dominant subtype. After multivariate adjustment, higher depressive symptom scores were positively associated with anxiety (OR = 1.386), whereas better sleep quality (OR = 0.965) and intact olfactory identification (OR = 0.815) were negatively associated with anxiety; motor severity showed no significant association after multivariable adjustment. Exploratory subgroup analyses revealed heterogeneous patterns of sleep and olfactory correlates across clinical strata. The three-factor predictive model yielded an overall classification accuracy of 84.81%.

**Conclusion:**

These findings indicate that non-motor abnormalities, rather than motor impairment, are associated with anxiety in early PD after adjustment for demographic and motor covariates. The distinct correlative patterns across subgroups provide preliminary clinical clues for the heterogeneous neurodegenerative processes underlying PD-related anxiety, and support routine screening of depressive, sleep and olfactory symptoms to facilitate anxiety risk stratification at initial diagnosis.

## 1 Introduction

Parkinson’s disease (PD) is a progressive neurodegenerative disorder defined by nigrostriatal dopaminergic degeneration, accompanied by a broad array of non-motor manifestations that compromise patients’ daily function and quality of life(Kalia and Lang, 2015; Lo Buono et al., 2021). Anxiety is one of the most prevalent non-motor features of PD, with a documented prevalence of 40%–50% among affected patients, far exceeding anxiety rates in age-matched community populations (Mammen et al., 2025). The presence of anxious symptoms correlates with accelerated functional decline and heavier caregiving burden (Lo Buono et al., 2021). Anxiety and depressive manifestations frequently co-occur as PD-related non-motor symptoms, instead of representing distinct primary psychiatric illnesses (Abou Kassm et al., 2021).

Most prior studies exploring anxiety in PD have enrolled patients receiving long-term dopaminergic replacement therapy (Berman et al., 2016). In treated cohorts, anxiety is confounded by levodopa-related motor fluctuations, emotional distress during off-states, and cumulative motor impairment (Blundell et al., 2023). These confounding factors complicate the differentiation between anxiety linked to early intrinsic PD pathology and secondary emotional distress induced by medication or advanced physical disability (Pontone et al., 2009). *De novo* PD patients eliminate these interferences, allowing clearer characterization of anxiety phenotypes associated with early neurodegenerative changes (Leodori et al., 2024).

Current evidence regarding the clinical correlates of anxiety in *de novo* PD remains incomplete. While univariate analyses have identified bivariate associations between anxiety and depressive symptoms, sleep disturbance, and olfactory dysfunction (Rutten et al., 2017; Abou Kassm et al., 2021), most existing studies have not applied comprehensive multivariate adjustment for demographic and motor covariates to disentangle adjusted clinical correlates. Furthermore, systematic stratified analyses across age, sex, disease duration and disease stage have not been consistently conducted in well-characterized, homogeneous *de novo* PD cohorts, which limits the development of targeted screening strategies for *de novo* patients. Given the heterogeneity in pathological burden, hormonal profiles and disease trajectories across patient subgroups, the patterns of association may differ across population strata.

Against this backdrop, this cross-sectional study was conducted within a well-characterized cohort of *de novo* PD patients, minimizing confounding from dopaminergic therapy and advanced disease pathology. We had three predefined objectives: (1) describe the phenotypic features of anxiety at initial PD diagnosis; (2) identify non-motor correlates of anxiety after adjusting for motor and demographic confounders; (3) explore potential subgroup differences in these correlative relationships across age, sex and disease strata. We hypothesized that non-motor manifestations, which reflect early neurodegenerative changes in extra-nigral pathways, may constitute stronger correlates of anxiety than motor severity after multivariable adjustment, and that these associations may vary across subgroups due to differing pathological burdens and aging-related neurobiological changes.

## 2 Patients and methods

### 2.1 Study participants

This retrospective cross-sectional study recruited consecutive *de novo* Parkinson’s disease (PD) patients from the Movement Disorders Unit, Affiliated Brain Hospital of Nanjing Medical University (October 2018–October 2022). Age- and sex-matched healthy volunteers served as controls.

All PD patients met the 2015 Movement Disorder Society (MDS) clinical diagnostic criteria for idiopathic PD (Postuma et al., 2015). Inclusion criteria were as follows: aged 45–75 years; no prior use of any antiparkinsonian medications (including levodopa, dopamine agonists, MAO-B inhibitors, COMT inhibitors, anticholinergics, and amantadine) or psychotropic medications (antidepressants, anxiolytics, sedative-hypnotics) at baseline; capacity to complete all standardized clinical assessments; minimum 1-year clinical follow-up (extended to 2 years for patients with baseline disease duration < 1 year). Patients who failed to meet idiopathic PD diagnostic criteria during follow-up were excluded.

Uniform exclusion criteria applied to all participants: intracranial structural lesions, other neurodegenerative or primary psychiatric disorders, secondary parkinsonism, Parkinson-plus syndromes, cognitive impairment defined by Chinese normative cut-offs of the Mini-Mental State Examination (MMSE) (Li et al., 2016), severe systemic diseases, and use of any neuroactive medications within 3 months prior to assessment. Additional exclusion criteria for controls included a first-degree family history of neurodegenerative disease, self-reported persistent olfactory or sleep dysfunction, and long-term use of neuroactive medications.

The study protocol was approved by the Medical Ethics Committee of the Affiliated Brain Hospital of Nanjing Medical University (approval No. 2019-KY019-01). All participants provided written informed consent prior to assessment, in accordance with the Declaration of Helsinki.

### 2.2 Clinical assessments

All clinical evaluations were administered by uniformly trained movement disorder neurologists in standardized quiet environments. Anxiety severity was measured with the 14-item Hamilton Anxiety Rating Scale (HAMA). Based on validated Chinese cut-off values, patients with a total HAMA score ≥ 7 were classified as PD with anxiety (A-PD), while those scoring below 7 were defined as PD without anxiety (NA-PD) (Luo et al., 2014).

Motor function was assessed using Parts II and III of the Unified Parkinson’s Disease Rating Scale (UPDRS), combined with Hoehn-Yahr (H-Y) staging (Liu et al., 2024); the Jankovic ratio method was applied to differentiate tremor-dominant, postural instability and gait difficulty (PIGD)-dominant, and mixed motor subtypes (Stebbins et al., 2013). Supplementary non-motor measurements included the MMSE for global cognitive function, the 24-item Hamilton Depression Rating Scale (HAMD-24) to quantify depressive manifestations, and the Parkinson’s Disease Sleep Scale (PDSS) for sleep quality. Olfactory identification performance was evaluated via the 12-item Sniffin’ Sticks test, with a total score ≤ 8 designated as hyposmia (Hummel et al., 2007).

### 2.3 Statistical analysis

All statistical analyses were conducted using SPSS 27.0, with a two-tailed significance level set at α = 0.05. Normally distributed continuous variables were presented as mean ± standard deviation and compared via independent *t*-tests or one-way analysis of variance (ANOVA). Non-normal data were reported as median (interquartile range, IQR) and analyzed with the Mann–Whitney U or Kruskal–Wallis H test. Categorical data were summarized as counts (percentages) and compared using the χ^2^ test or Fisher’s exact test where appropriate. Bonferroni correction was applied for post-hoc pairwise comparisons to control type I error.

Bidirectional stepwise binary logistic regression (entry criterion *p* < 0.05, removal criterion *p* > 0.10) was used to identify correlates of anxiety after adjustment for all prespecified demographic and motor covariates. Model calibration was evaluated by the Hosmer-Lemeshow test; discriminative performance was quantified using Nagelkerke *R*^2^ and overall classification accuracy. Supplementary sensitivity analyses were performed to address scale item overlap and stepwise selection bias.

Prespecified stratified analyses were performed by age, sex, disease duration, H-Y stage and UPDRS-III severity. All subgroup analyses were interpreted as exploratory without correction for multiple comparisons, and this limitation will be addressed in the Discussion section.

Sample size was calculated based on 40%–50% expected anxiety prevalence, 5% margin of error and 95% confidence interval, targeting 250–350 PD patients. The final cohort included 284 *de novo* PD patients with 125 anxiety events, yielding 18–25 events per predictor, satisfying the ≥10 events-per-variable rule for stable logistic regression.

## 3 Results

### 3.1 Baseline demographic and clinical characteristics

A total of 284 *de novo* PD patients and 147 age- and sex-matched healthy controls were enrolled. According to the HAMA cut-off value of 7, 125 patients (44.0%) were classified into the A-PD subgroup with clinical anxiety, and 159 (56.0%) into the NA-PD subgroup. Anxiety prevalence was significantly higher in PD patients than in healthy controls (*p* < 0.001).

Age, sex distribution and educational years were comparable across all three groups (all *p* > 0.05). No significant differences in age at onset or disease duration were found between A-PD and NA-PD groups. Compared with NA-PD patients, the A-PD subgroup had higher HAMD-24 scores, lower PDSS scores, poorer olfactory identification performance and a higher rate of hyposmia (all *p* < 0.001). MMSE scores did not differ significantly between cohorts. Full baseline data are presented in Table 1.

**Table 1:** Baseline characteristics of study cohorts.

Variable	A-PD (*n* = 125)	NA-PD (*n* = 159)	Controls (*n* = 147)	Statistic	*p*-value
Age, years, mean ± SD	60.22 ± 8.04	58.93 ± 8.89	60.46 ± 7.29	*F* = 1.560	0.211
Male, *n* (%)	51 (40.8)	79 (49.7)	67 (45.6)	*χ^2^* = 2.228	0.328
Education, years, median (IQR)	9.0 (7.0, 12.0)	10.0 (7.7, 12.0)	10.0 (8.0, 12.0)	*H* = 1.203	0.548
Age at onset, years, mean ± SD	58.08 ± 8.42	57.02 ± 9.17	NA	*t* = 0.998	0.319
Disease duration, years, median (IQR)	2.0 (1.0, 3.0)	1.0 (1.0, 3.0)	NA	*z* = −1.544	0.123
HAMA total, median (IQR)	10.0 (8.0, 13.0)	3.0 (1.0, 5.0)	1.0 (0, 4.0)	*H* = 258.952	<0.001
HAMD-24 total, median (IQR)	14.0 (11.0, 18.5)	5.0 (3.0, 8.0)	1.0 (0, 4.0)	*H* = 253.589	<0.001
MMSE total, median (IQR)	28.0 (28.0, 29.0)	29.0 (28.0, 29.0)	29.0 (28.0, 30.0)	*H* = 1.565	0.503
PDSS total, median (IQR)	118.0 (101.0, 131.0)	139.0 (130.0, 144.0)	NA	*z* = −9.257	<0.001
Olfactory score, median (IQR)	6.0 (5.0, 7.0)	7.0 (6.0, 9.0)	NA	*z* = −5.619	<0.001
Hyposmia, *n* (%)	104 (83.2)	87 (54.7)	NA	*χ^2^* = 25.780	<0.001

Data are presented as mean ± standard deviation, median (interquartile range), or n (%). A-PD, PD with anxiety; NA-PD, PD without anxiety; HC, healthy controls; NA, not applicable; HAMA, Hamilton Anxiety Rating Scale; HAMD-24, 24-item Hamilton Depression Rating Scale; MMSE, Mini-Mental State Examination; PDSS, Parkinson’s Disease Sleep Scale.

### 3.2 Phenotypic distribution of anxiety symptoms

Psychic anxiety constituted the dominant symptom subtype in A-PD patients, while no significant difference between psychic and somatic subscores was observed in the NA-PD group. The prevalence of all 14 individual HAMA items was significantly higher in A-PD patients (all *p* < 0.001). Detailed subscale comparisons are summarized in Table 2 and Figure 1.

**Table 2:** HAMA total and subscale scores in A-PD and NA-PD subgroups.

Group	*n* (%)	Total score	Psychic anxiety subscore	Somatic anxiety subscore	*z*-statistic*	*p*-value
A-PD	125 (44.0)	10.0 (8.0, 13.0)	7.0 (5.0, 9.0)	4.0 (3.0, 5.0)	−7.978	< 0.001
NA-PD	159 (56.0)	3.0 (1.0, 5.0)	1.0 (0, 3.0)	1.0 (0, 2.0)	−1.695	0.090

Data are presented as median (interquartile range) or *n* (%). *Within-group comparison of psychic vs somatic subscore, Wilcoxon signed-rank test.

A-PD, PD with anxiety; NA-PD, PD without anxiety.

**Figure 1:**
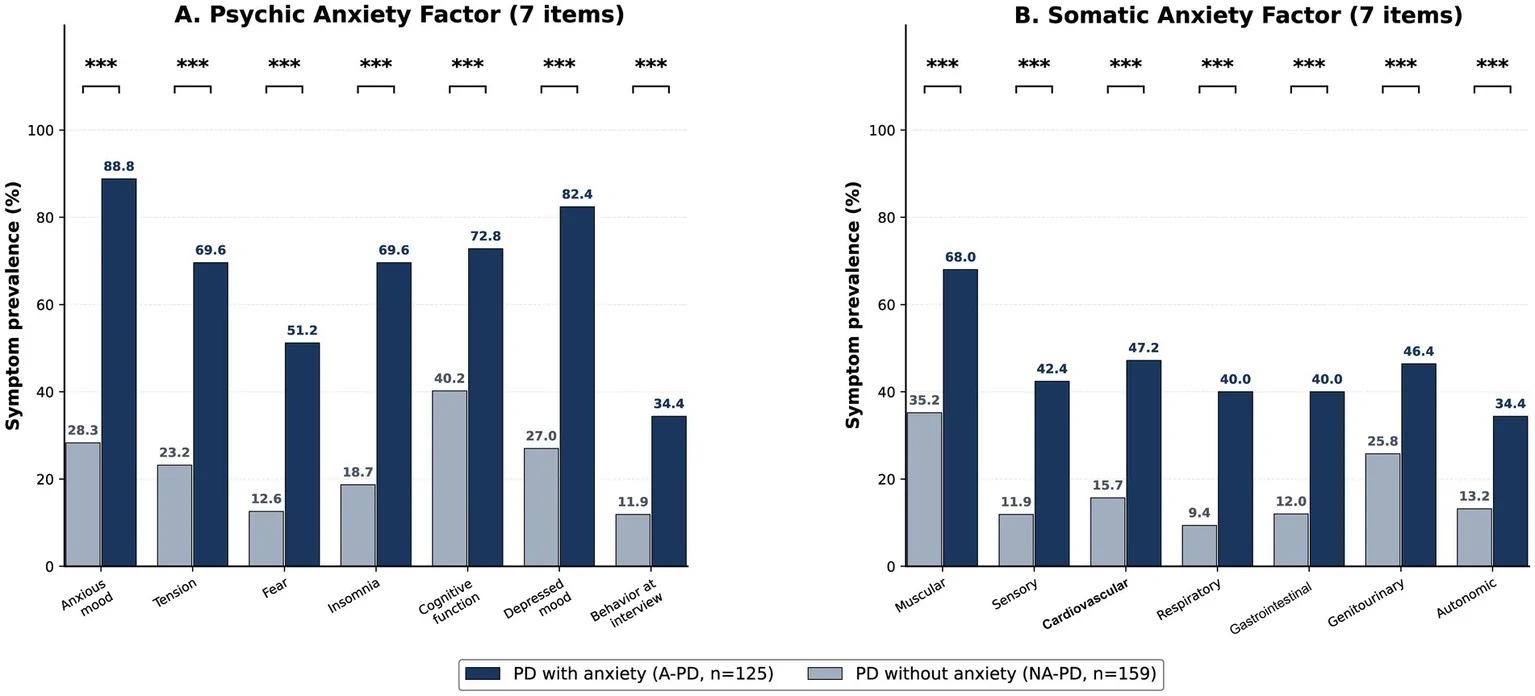
Symptom prevalence across 14 HAMA items stratified by anxiety status in *de novo* PD. Panels **(A)** and **(B)** display the seven items of the Psychic Anxiety Factor and the seven items of the Somatic Anxiety Factor, respectively. Bars represent symptom prevalence (%) in PD with anxiety (A-PD, *n* = 125) and PD without anxiety (NA-PD, *n* = 159). ***p < 0.001 for all 14 comparisons (A-PD vs. NA-PD) by chi-square test. HAMA = Hamilton Anxiety Rating Scale.

### 3.3 Between-group comparisons of motor manifestations

Univariate analysis showed more severe overall motor impairment in A-PD patients, mainly reflected in worse postural-gait dysfunction and bradykinesia (both *p* < 0.05). Tremor and rigidity severity, as well as the distribution of the three motor subtypes, did not differ significantly between groups. A higher proportion of A-PD patients had H-Y stage > 2.5 (*p* = 0.001). All motor-related comparative data are listed in Table 3.

**Table 3:** Motor severity comparisons between A-PD and NA-PD.

Variable	A-PD (*n* = 125)	NA-PD (*n* = 159)	Statistic	*p*-value
UPDRS-III total, median (IQR)	22.0 (16.0, 29.5)	18.0 (13.0, 25.0)	*z* = −3.381	<0.001
Tremor subscore	2.0 (1.0, 4.5)	2.0 (1.0, 3.0)	*z* = −1.307	0.191
Rigidity subscore	5.0 (2.0, 7.0)	4.0 (2.0, 6.0)	*z* = −1.944	0.052
Postural-gait subscore	3.0 (2.0, 3.5)	2.0 (1.0, 3.0)	*z* = −3.494	<0.001
Bradykinesia subscore	10.0 (7.0, 16.0)	9.0 (6.0, 13.0)	*z* = −2.359	0.018
H-Y stage, median (IQR)	2.0 (1.5, 2.3)	1.5 (1.0, 2.0)	*z* = −3.863	<0.001
H-Y > 2.5, *n* (%)	31 (24.8)	16 (10.1)	*χ^2^* = 11.005	0.001
Motor subtype, *n* (%)			*χ^2^* = 1.218	0.544
Tremor-dominant	53 (42.4)	72 (45.3)		
PIGD-dominant	7 (5.6)	13 (8.2)		
Mixed	65 (52.0)	74 (46.5)		

Data are presented as median (interquartile range) or *n* (%).

UPDRS-III, Unified Parkinson’s Disease Rating Scale Part III; PIGD, postural instability and gait difficulty; H-Y, Hoehn-Yahr; A-PD, PD with anxiety; NA-PD, PD without anxiety.

### 3.4 Non-motor correlates of anxiety after multivariable adjustment

All prespecified candidate variables, including demographic factors (age, sex, education, disease duration), motor severity markers (H-Y stage, UPDRS-III total score), and non-motor factors, were entered into the initial variable pool for bidirectional stepwise logistic regression. After stepwise selection, three non-motor indicators remained associated with anxiety in the final model, while all motor severity measures failed to meet the entry threshold and were excluded from the final model (Table 4). Higher HAMD-24 scores were positively associated with elevated anxiety odds (OR = 1.386, 95% CI 1.264–1.520, *p* < 0.001). Higher PDSS scores (indicating better sleep quality) and better olfactory identification were inversely associated with anxiety odds, with OR values of 0.965 (95% CI 0.941–0.990, *p* = 0.007) and 0.815 (95% CI 0.680–0.977, *p* = 0.027), respectively.

**Table 4:** Multivariate stepwise logistic regression of correlates of anxiety.

Predictor	*β*	SE	Wald χ^2^	*p*-value	OR	95% CI
HAMD-24 score	0.326	0.047	48.068	<0.001	1.386	1.264–1.520
PDSS score	−0.036	0.013	7.350	0.007	0.965	0.941–0.990
Olfactory score	−0.204	0.093	4.868	0.027	0.815	0.680–0.977
Intercept	2.562	1.905	1.808	0.179	12.959	0.310–542.446

Hosmer–Lemeshow: χ^2^ = 13.256, *p* = 0.103; Nagelkerke R^2^ = 0.653; accuracy = 84.81%.

HAMD-24, 24-item Hamilton Depression Rating Scale; PDSS, Parkinson’s Disease Sleep Scale; OR, odds ratio; CI, confidence interval.

The regression model showed acceptable goodness-of-fit (Hosmer-Lemeshow *χ^2^* = 13.256, *p* = 0.103), with a Nagelkerke R^2^ of 0.653 and an overall classification accuracy of 84.81%.

### 3.5 Sensitivity analyses

Supplementary sensitivity analyses were conducted to address methodological concerns. First, for potential scale item overlap between HAMD-24 and HAMA, Spearman correlation confirmed a strong association between total scores (*r* = 0.815, *p* < 0.001), but collinearity diagnostics showed all variance inflation factor (VIF) values were below 2 (HAMD-24: VIF = 1.832), indicating no severe multicollinearity. After removing overlapping items and re-fitting the stepwise model, the adjusted depressive symptom score remained highly significantly associated with anxiety (OR = 1.362, 95% CI 1.260–1.472, *p* < 0.001), and olfactory identification remained a significant protective factor (OR = 0.851, 95% CI 0.726–0.997, *p* = 0.045); the PDSS sleep score was attenuated and did not meet the entry threshold. Second, a fully adjusted logistic regression using the enter method with all nine prespecified covariates yielded nearly identical results to the stepwise model, confirming that core findings are robust and not an artifact of variable selection. Detailed results are provided in Supplementary Tables 1–3.

### 3.6 Exploratory subgroup heterogeneity

All subgroup analyses were prespecified exploratory analyses and were not adjusted for multiple comparisons; results are intended solely for hypothesis generation. Exploratory stratified analyses revealed tentative heterogeneous correlative patterns across subgroups (Table 5; Figure 2). Depressive manifestations were consistently associated with higher anxiety odds across all strata. Olfactory impairment was only significantly associated with anxiety in males, patients aged ≥ 60 years, and those with H-Y stage > 2.5. Poor sleep was significantly associated with anxiety in females, patients aged under 60 years, those with disease duration ≥ 2 years and those at earlier H-Y stages, as well as across both motor severity strata. Longer educational attainment was a significant correlate of higher anxiety odds in patients younger than 60 years and those with disease duration ≥ 2 years.

**Table 5:** Subgroup-specific correlates of anxiety (exploratory stepwise logistic regression).

Stratification	Subgroup	Factor	OR (95% CI)	*p*-value
Age	<60 years	Education level	1.152 (1.009–1.314)	0.036
		HAMD-24	1.349 (1.191–1.529)	<0.001
		PDSS	0.942 (0.902–0.983)	0.006
	≥60 years	HAMD-24	1.551 (1.342–1.793)	<0.001
		Olfactory identification	0.762 (0.594–0.977)	0.032
Sex	Male	HAMD-24	1.379 (1.224–1.553)	<0.001
		Olfactory identification	0.706 (0.528–0.944)	0.019
	Female	HAMD-24	1.467 (1.276–1.686)	<0.001
		PDSS	0.954 (0.920–0.989)	0.010
Disease duration	<2 years	HAMD-24	1.485 (1.305–1.689)	<0.001
	≥2 years	Education level	1.174 (1.027–1.342)	0.019
		HAMD-24	1.390 (1.208–1.599)	<0.001
		PDSS	0.928 (0.889–0.968)	<0.001
H-Y stage	≤2.5	HAMD-24	1.475 (1.280–1.700)	<0.001
		PDSS	0.951 (0.913–0.991)	0.016
	>2.5	HAMD-24	1.381 (1.231–1.549)	<0.001
		Olfactory identification	0.737 (0.576–0.941)	0.015
UPDRS-III	≤20	HAMD-24	1.507 (1.304–1.742)	<0.001
		PDSS	0.961 (0.926–0.998)	0.038
	>20	HAMD-24	1.320 (1.169–1.491)	<0.001
		PDSS	0.960 (0.927–0.994)	0.021

OR, odds ratio; CI, confidence interval. A *p*-value < 0.05 was considered statistically significant. All subgroup analyses are prespecified exploratory analyses and have not been adjusted for multiple comparisons. Results are hypothesis-generating only and require validation in larger independent cohorts.

UPDRS-III, Unified Parkinson’s Disease Rating Scale Part III; H-Y, Hoehn-Yahr; PDSS, Parkinson’s Disease Sleep Scale; HAMD-24, 24-item Hamilton Depression Rating Scale.

**Figure 2:**
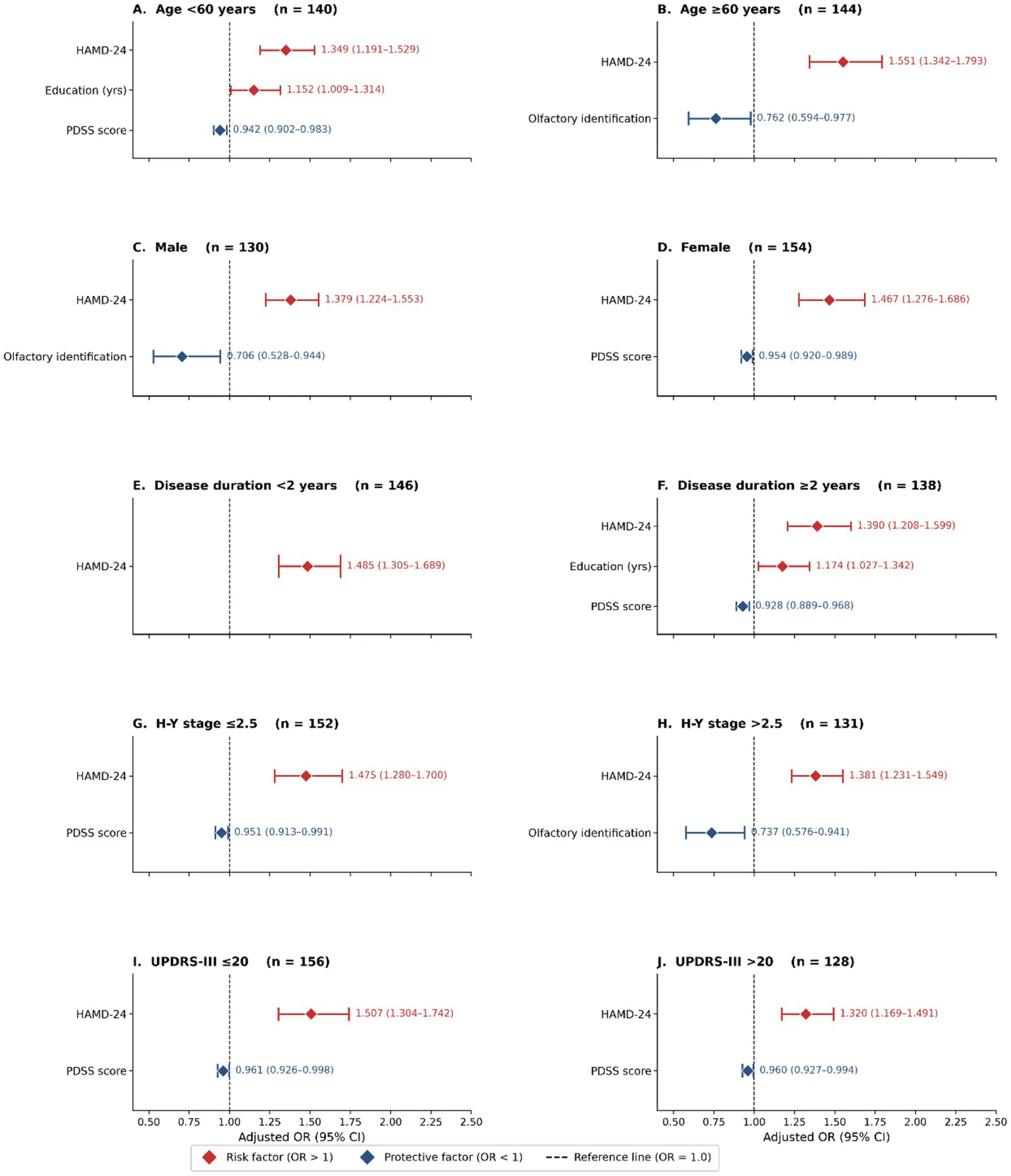
Subgroup analysis of factors associated with anxiety in *de novo* PD. Panels **(A–J)** correspond to 10 subgroups across 5 prespecified stratification dimensions: **(A)** Age < 60 years, **(B)** Age ≥ 60 years, **(C)** Male, **(D)** Female, **(E)** Disease duration < 2 years, **(F)** Disease duration ≥ 2 years, **(G)** H-Y stage ≤ 2.5, **(H)** H-Y stage > 2.5, **(I)** UPDRS-III ≤ 20, **(J)** UPDRS-III > 20. Adjusted odds ratios (ORs) with 95% confidence intervals were derived from multivariable logistic regression models within each prespecified subgroup. Diamonds represent OR point estimates; horizontal lines represent 95% CIs. Red = risk factors (OR > 1); Blue = protective factors (OR < 1); Dashed line = reference (OR = 1.0). All subgroup analyses are prespecified exploratory analyses and have not been adjusted for multiple comparisons. Results are hypothesis-generating only. H-Y, Hoehn-Yahr; UPDRS-III, Unified Parkinson’s Disease Rating Scale part III; HAMD-24, 24-item Hamilton Depression Rating Scale; PDSS, Parkinson’s Disease Sleep Scale; OR, odds ratio; CI, confidence interval.

## 4 Discussion

As a prevalent age-related neurodegenerative disorder, PD presents with a broad spectrum of non-motor symptoms that often precede overt motor impairment and reflect early extra-nigral neurodegeneration. These non-motor features have become major determinants of impaired quality of life in affected patients. Anxiety is one of the most common early non-motor symptoms of PD, but its clinical correlates and underlying neurobiological mechanisms in the *de novo* stage remain incompletely understood. This study focused on newly diagnosed, medication-free PD patients, a design that eliminates confounding from dopaminergic therapy and advanced disease pathology. Our findings not only characterize non-motor correlates after multivariable adjustment of anxiety in early PD, but also provide indirect clinical clues for the potential involvement of serotonergic, noradrenergic and olfactory-limbic pathway degeneration.

In this cohort, the screening-positive rate of anxiety (HAMA total score ≥ 7) was 44.0% in *de novo* PD patients, significantly higher than that in healthy controls. Psychic anxiety was the dominant subtype, with the highest prevalence observed for anxious mood, depressive mood, and anxiety-related cognitive items. This finding is consistent with previously reported co-occurrence of depression and anxiety in PD populations (Tiller, 2013; Zhao et al., 2025), further supporting that this phenotype is prevalent even in early-stage disease. It also indicates that PD-related anxiety occurs early in the disease course and may be closely associated with primary pathological alterations of PD (Hasbi et al., 2020; Morel et al., 2022), rather than being purely secondary to disease burden or pharmacotherapy.

Stepwise logistic regression analysis in this study revealed that depressive symptoms, sleep quality, and olfactory identification ability were correlates of anxiety after multivariable adjustment in *de novo* PD patients, whereas motor severity showed no significant association after adjustment. This finding suggests that pathophysiological changes in the non-motor domain play an important role in the development of anxiety in early PD, which differs from conclusions of most studies focusing on anxiety in advanced PD. This discrepancy may arise because anxiety in advanced patients is more susceptible to secondary factors such as adverse drug effects and severe motor deterioration (Cilia et al., 2020; Hommel et al., 2020).

The present study found that depressive symptoms were the strongest correlate of anxiety after multivariable adjustment (OR = 1.386), and this association remained robust across all clinical subgroups. This high comorbidity is consistent with extensive prior clinical and neuropathological evidence. A well-supported explanation is that early degeneration of serotonergic neurons in the dorsal raphe nucleus — a core extra-nigral site affected in the early Braak stages of PD (Braak et al., 2003; Maillet et al., 2016; Nobis et al., 2023) — leads to widespread serotonergic innervation deficits in the limbic system and prefrontal cortex. Combined with impaired mesolimbic dopaminergic transmission (Watabe-Uchida et al., 2012; Monzani et al., 2019) and prefrontal-amygdala circuitry dysregulation (Carey et al., 2021; Zhang et al., 2022), these age-related neurodegenerative changes may collectively drive the co-occurrence of depression and anxiety in early-stage PD, which aligns with the strong association observed in our *de novo* cohort.

We observed that better sleep quality was significantly associated with lower odds of anxiety (OR = 0.965). The locus coeruleus, the primary source of cerebral noradrenaline, is one of the earliest sites of α-synuclein pathology in PD (Braak et al., 2003), and its degeneration is exacerbated by aging (Feinstein et al., 2016). Noradrenergic projections from the locus coeruleus widely regulate both sleep–wake architecture and amygdala-mediated fear responses (Itoi, 2008). Therefore, early locus coeruleus impairment may serve as a shared pathological substrate linking sleep disturbance and anxiety in *de novo* PD, consistent with the correlative pattern observed in our cohort.

Intact olfactory identification was significantly associated with lower anxiety risk after multivariable adjustment (OR = 0.815). Olfactory impairment is a well-recognized early non-motor marker of PD, corresponding to α-synuclein deposition in the olfactory bulb and anterior olfactory nucleus — the putative starting point of Braak pathological staging (Braak et al., 2003). Olfactory structures have extensive reciprocal connections with limbic regions including the amygdala and entorhinal cortex. Thus, progressive α-synuclein pathology along the olfactory-limbic axis may simultaneously impair olfactory function and promote emotional dysregulation (Socała et al., 2021; Needham et al., 2022). This topographical alignment with Braak staging suggests that the olfactory-anxiety association may reflect greater overall neurodegenerative burden in older patients with more advanced disease stage, consistent with our subgroup findings.

Exploratory stratified analyses showed heterogeneous non-motor correlates of anxiety across subgroups, potentially reflecting varying neurodegenerative pathology burdens. Depressive symptoms were universally associated with anxiety across all strata, consistent with shared serotonergic-limbic dysfunction and neurocircuitry models (Treadway, 2025). Olfactory impairment correlated with anxiety only in older, male and more advanced-stage patients, possibly driven by age-related pathology progression (Feinstein et al., 2016), sex hormone effects (Sullivan et al., 2014; Wang et al., 2017; Cao et al., 2022) and Braak-stage limbic involvement (Braak et al., 2003). Sleep quality correlated with anxiety mainly in females, longer-duration and earlier-stage patients, likely via sex-differentiated noradrenergic stress responses (Bates et al., 2023; Hodes et al., 2024) and shared locus coeruleus-mesolimbic pathway damage (Feinstein et al., 2016; Morel et al., 2022). Educational attainment correlated with anxiety in younger, longer-duration patients, probably due to greater disease awareness and anticipatory rumination (Donohue et al., 2024; van der Heide et al., 2024). All subgroup results are exploratory and need further multicenter validation.

The principal strengths of this study include the well-characterized *de novo* PD cohort free from dopaminergic medication confounding, comprehensive multi-domain non-motor assessments, systematic exploratory subgroup analyses, and rigorous diagnostic validation via longitudinal specialist follow-up. Several limitations should be acknowledged. First, the cross-sectional design precludes causal inference; longitudinal studies are needed to establish temporal relationships between non-motor indicators and anxiety in early PD. Second, the single-center cohort may limit generalizability to other populations. Third, all assessments were based on clinical rating scales without cerebrospinal fluid or neuroimaging biomarkers; thus, the neuropathological interpretations discussed above are derived from existing literature and cannot be directly verified by the present data. Partial item overlap between the HAMD-24 and HAMA scales may also introduce common method variance. Although sensitivity analyses confirmed the robustness of the core findings, future studies using more specific biomarkers or objective assessment tools are needed for validation. Fourth, stepwise variable selection has known limitations, including potential overestimation of effect sizes. We mitigated this risk using conservative entry/removal thresholds and validation with a fully adjusted enter model, but prospective longitudinal studies are still needed to establish causality. Fifth, dopamine transporter imaging was not routinely performed, though diagnosis by movement disorder specialists and 1–2 years of longitudinal follow-up with treatment response assessment largely mitigated the risk of diagnostic misclassification. Sixth, subgroup analyses were exploratory with limited stratum sample sizes; the observed heterogeneity requires confirmation in larger multicenter cohorts. Finally, the classification model was only internally validated, and external validation is needed for clinical application.

## 5 Conclusion

Depressive symptoms, poor sleep quality and olfactory dysfunction are non-motor correlates of anxiety in *de novo* PD after multivariable adjustment, whereas motor severity shows no significant association after adjustment. Exploratory analyses reveal tentative heterogeneous correlative patterns of sleep and olfactory function with anxiety across sex, age and disease stage strata, which may reflect differing trajectories of early neurodegeneration. These findings suggest the potential involvement of extra-nigral non-motor pathways in early PD-related anxiety, and support the integration of depressive, sleep and olfactory assessments into routine initial evaluation to facilitate anxiety risk stratification and targeted management.

## Data Availability

The original contributions presented in the study are included in the article/Supplementary material, further inquiries can be directed to the corresponding authors.
