# Supplementary material for: Non-motor correlates of anxiety in de novo Parkinson’s disease: a cross-sectional study with exploratory subgroup analyses
Source: Front Neurosci. 2026 Sep 25;20:1955684. doi: 10.3389/fnins.2026.1955684 (PMC13650010; doi:10.3389/fnins.2026.1955684)
Supplement: Supplementary file 1 [file Table_1.docx]

Supplementary Table 1. Collinearity diagnostics for the full multivariable model

| **Variable** | **VIF** | **Tolerance** |
| --- | --- | --- |
| HAMD-24 | 1.832 | 0.546 |
| Age | 1.069 | 0.935 |
| Sex | 1.045 | 0.957 |
| Disease duration | 1.019 | 0.981 |
| H-Y stage | 1.577 | 0.634 |
| UPDRS-III | 1.536 | 0.651 |
| PDSS | 1.701 | 0.588 |
| Olfactory identification | 1.113 | 0.899 |

*Note.* All VIF values are below 2, well below the conventional threshold of 10, indicating no severe multicollinearity.

VIF, variance inflation factor; HAMD-24, 24-item Hamilton Depression Rating Scale; H-Y, Hoehn-Yahr; UPDRS-III, Unified Parkinson's Disease Rating Scale part III; PDSS, Parkinson's Disease Sleep Scale.

Supplementary Table 2. Stepwise logistic regression results after removal of overlapping scale items

| **Variable** | **OR** | **95% CI** | ***p*-value** |
| --- | --- | --- | --- |
| Adjusted HAMD-24 score | 1.362 | 1.260–1.472 | <0.001 |
| Olfactory identification | 0.851 | 0.726–0.997 | 0.045 |
| Constant | – | – | <0.001 |

*Note.* Dependent variable: anxiety status defined by adjusted HAMA score. The PDSS sleep score did not meet the entry threshold and was not retained in the final model.

OR, odds ratio; CI, confidence interval; HAMD-24, 24-item Hamilton Depression Rating Scale;

Supplementary Table 3. Fully adjusted logistic regression results (forced entry method, robustness validation)

| **Variable** | **OR** | **95% CI** | ***p*-value** |
| --- | --- | --- | --- |
| HAMD-24 | 1.404 | 1.269–1.553 | <0.001 |
| PDSS | 0.965 | 0.939–0.992 | 0.010 |
| Olfactory identification | 0.816 | 0.679–0.982 | 0.031 |
| H-Y stage | 1.654 | 0.764–3.579 | 0.202 |
| UPDRS-III | 0.986 | 0.943–1.031 | 0.531 |
| Age | 1.007 | 0.961–1.056 | 0.764 |
| Sex | 0.986 | 0.461–2.109 | 0.970 |
| Disease duration | 1.013 | 0.878–1.170 | 0.855 |
| Education years | 1.079 | 0.986–1.180 | 0.099 |

*Note.* All covariates were entered simultaneously into the model. Core findings are highly consistent with the stepwise model.

OR, odds ratio; CI, confidence interval; HAMD-24, 24-item Hamilton Depression Rating Scale; PDSS, Parkinson's Disease Sleep Scale; H-Y, Hoehn-Yahr; UPDRS-III, Unified Parkinson's Disease Rating Scale part III.
